# Supplementary material for: Synucleinopathy alters nanoscale organization and diffusion in the brain extracellular space through hyaluronan remodeling
Source: Nat Commun. 2020 Jul 10;11:3440. doi: 10.1038/s41467-020-17328-9 (PMC7351768; doi:10.1038/s41467-020-17328-9)
Supplement: Supplementary file 1 — Supplementary Information [file 41467_2020_17328_MOESM1_ESM.pdf]

Supplementary Information

Synucleinopathy alters nanoscale organization and diffusion in the brain extracellular space through hyaluronan remodeling

Soria et al.

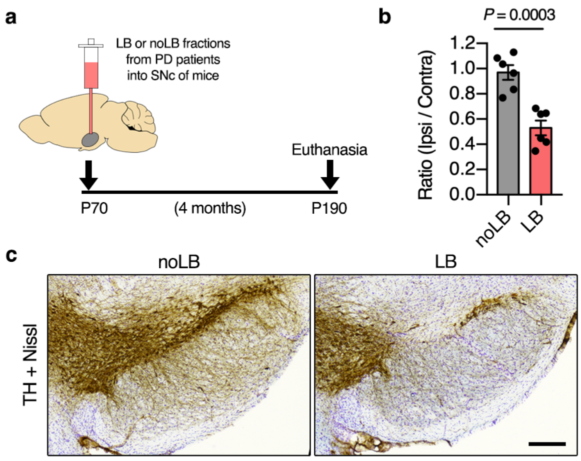

Supplementary Figure 1. Lewy body (LB) fractions from PD patients trigger nigral dopaminergic neurodegeneration in mice. *Related to Figure 1.* **a.** Experimental timeline of the LB-induced neurodegeneration model (described in detail in references 20 and 21). **b.** Ipsilateral/contralateral TH-positive cell ratio confirmed 40% dopaminergic cell loss in LB-injected mice after 4 months (Two-tailed Student's t test, n = 6 mice). **c.** TH staining shows dopaminergic neurodegeneration in the SN 4 months after injection of LB fractions from Parkinson's disease patients, which contain aggregated human alpha-synuclein. Inoculation of noLB fractions from the same patients, containing soluble non-aggregated alpha-synuclein, fails to convey neurodegeneration after 4 months in mice. Scale bar, 200 µm. Error bars represent mean ± SEM. Source data are provided as a Source Data file.

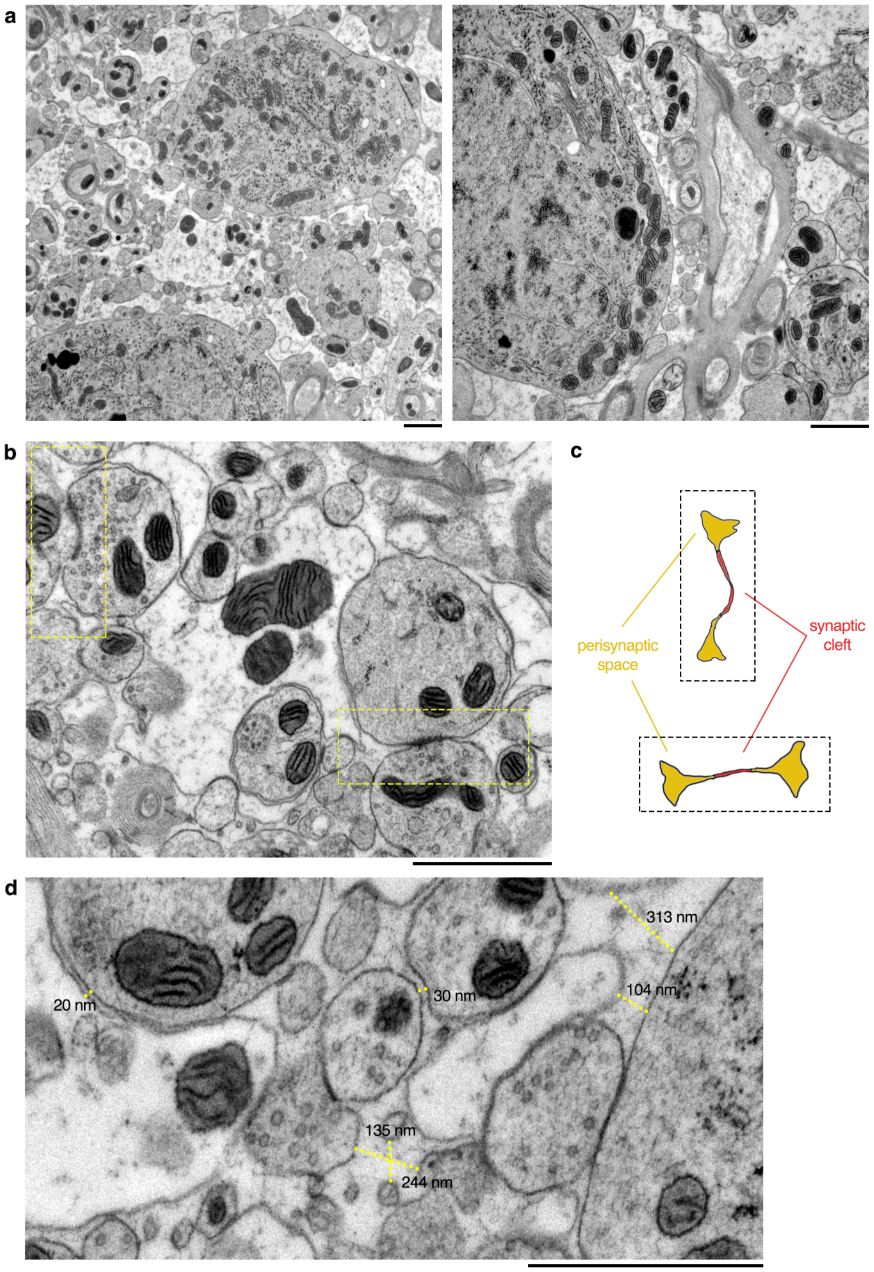

Supplementary Figure 2. High-pressure cryofixation provides a more accurate representation of the brain ECS. *Related to Figure 1.* **a.** Ultramicrographs of the SN neuropil after cryofixation, showing remarkable preservation of ECS dimensions, membrane and myelin structures. Cryofixation fixes molecules in their hydrated state, whereas freeze substitution resin embedding allows ultrathin sections in tissue where neuroanatomical structures retain their original hydrated positions. **b.** Ultramicrograph of cryofixed SN showing a phagocytic cell and two synapses. Cryofixation-EM retains large ECS compartments, such as the perisynaptic spaces, as well as small gaps like the synaptic cleft or the intermembrane space between the phagocytic and phagocyted cell. **c** Segmentation of the synapses depicted in (**b**). **d.** ECS widths are highly heterogeneous. Preservation of cellular membranes in cryofixation-EM allows for identification of narrow ECS gaps of 20-30 nm. Large ECS widths (> 200 nm) are frequent in the SN, as recently described for other brain structures. Scale bars, 1 µm.

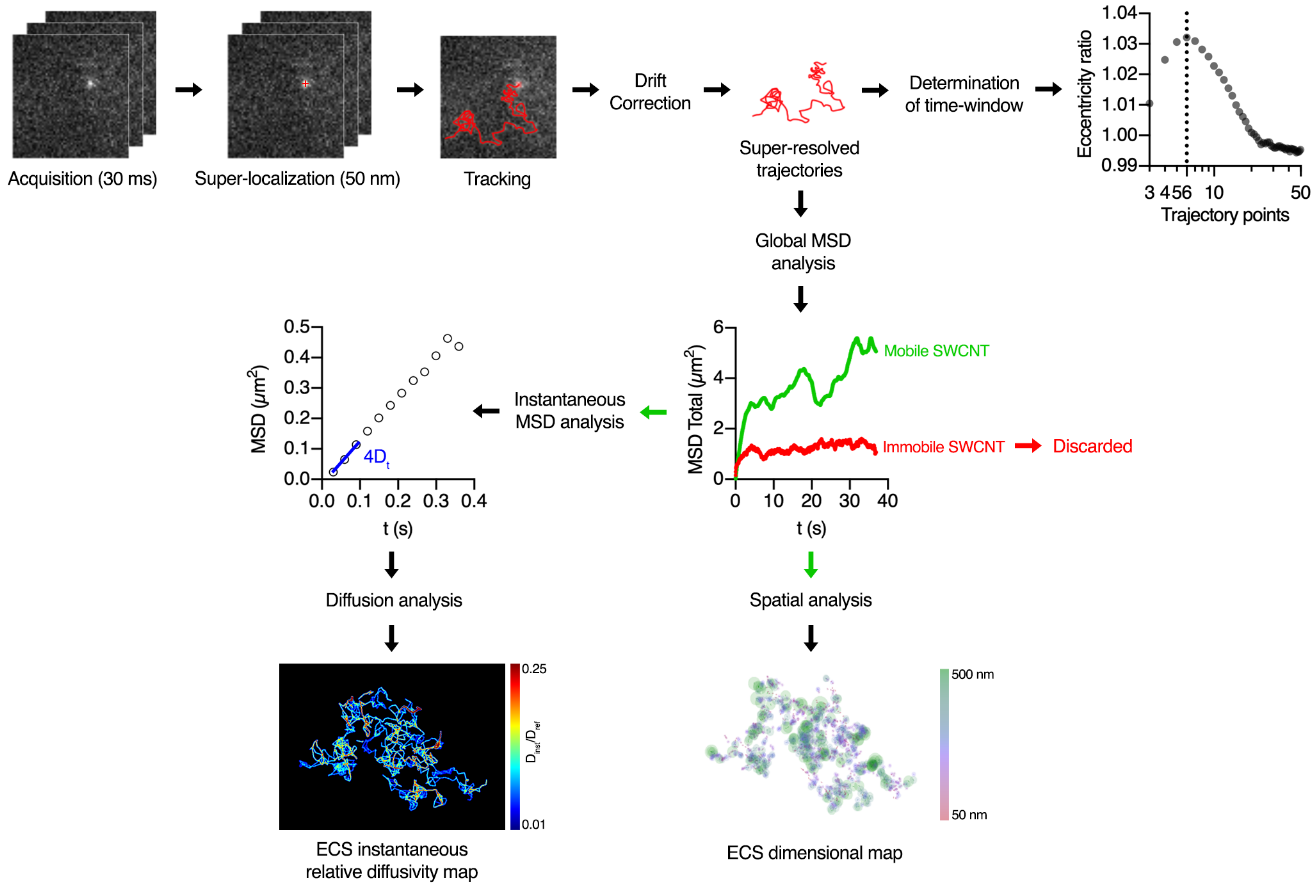

Supplementary Figure 3. Super-resolution image analysis protocol for single-nanotube tracking. *Related to Figure 2.* SWCNT images are acquired at a frame rate of 1/(30 ms) (total acquisition time = 5 min). SWCNT detections are averaged and fitted to obtain the super-resolved localization of the nanotube in each frame. Drift-corrected single-molecule tracks are used to determine the global mean square displacement (*MSD*). After discarding immobile SWCNTs (plateau-shaped *MSD*), instantaneous diffusion constants (D_{inst}) are calculated using a sliding window of 450 ms, and related to the theoretical free diffusion of the nanotube in CSF (D_{ref}). Local relative diffusivity maps are created based on the super-resolved trajectories previously calculated. Estimation of the ECS dimensions are based on the local geometric confinement experienced by the nanotube along its exploration. ECS width ξ is determined as the shortest dimension of the ellipse that describes the area explored by the nanotube in a 6-point trajectory. The size of the sliding window is determined by calculating the maximum eccentricity ratio (maximum confinement) of the ellipse describing the local ECS shape on super-resolved trajectories of increasing number of points (*upper right corner*).

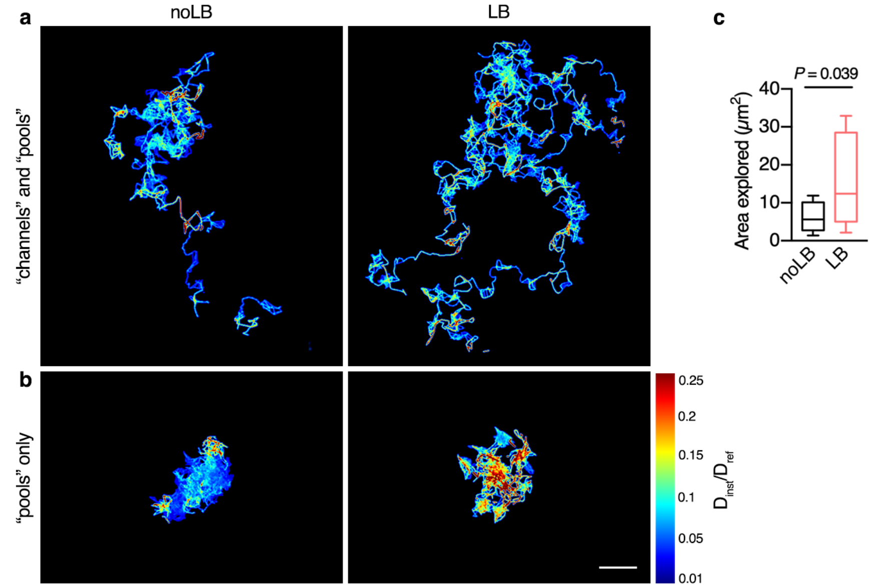

Supplementary Figure 4. **Heterogeneity of nanoscale diffusion in the brain ECS.** *Related to Figure 2***.** **a, b**. ECS local diffusivity maps reveal two types of SWCNT exploration that resemble the geometries observed by EM data. An abundance of channels that SWCNT visit a few times, connecting large pools where SWCNTs explore extensively. Trajectories reveal areas where the ECS shows a combination of both configurations (**a**) or regions where pools are predominant (**b**). Scale bar, 2 µm. **c.** Area covered by the SWCNT tracks. Note that this parameter is calculated from the super-resolved trajectories, hence they might underestimate the real area of the ECS compartment explored (two-tailed Student *t* test, *n* = 9 tracks). Boxes represent Q1, Q3 and median, whiskers represent min and max values. Source data are provided as a Source Data file.

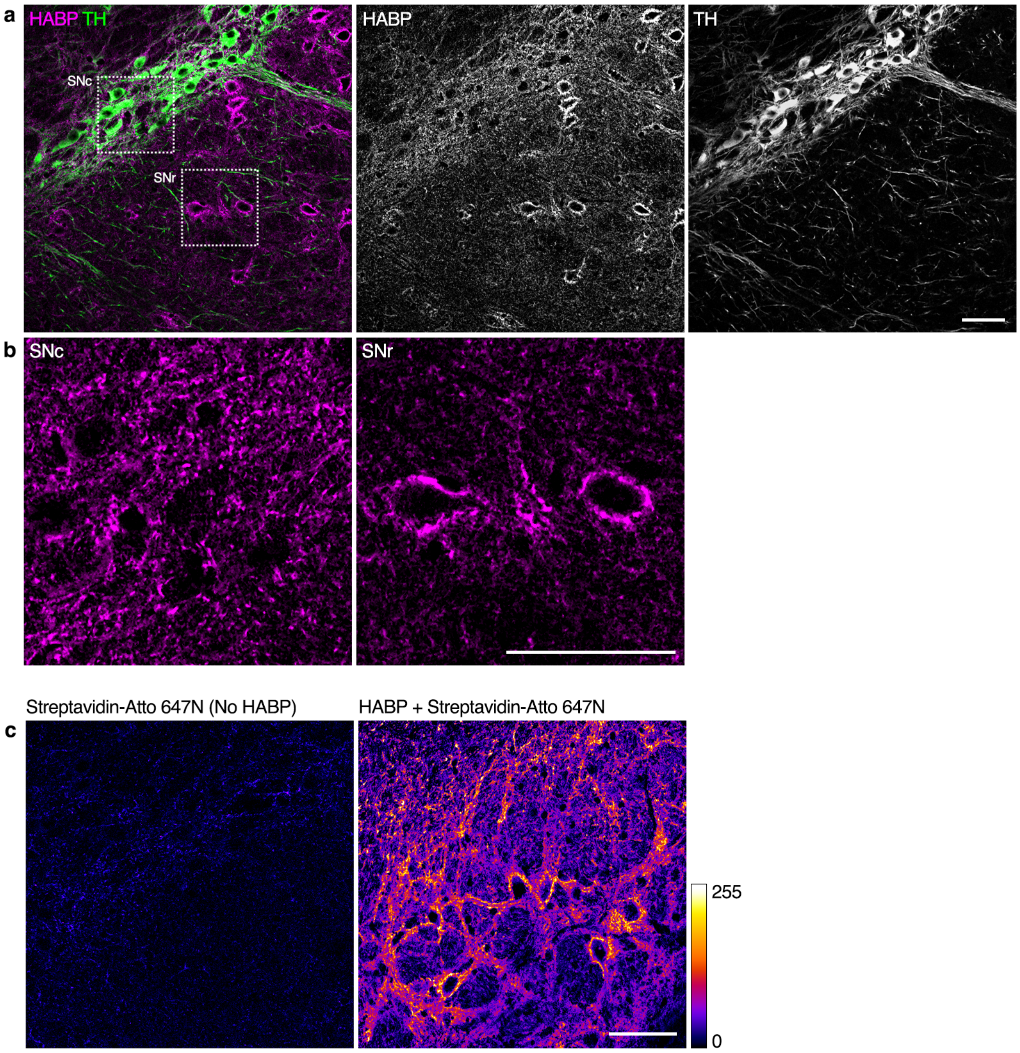

Supplementary Figure 5. Hyaluronan-binding protein (HABP) staining in the *substantia nigra* of mice. *Related to Figure 3*. **a.** Confocal micrographs showing HABP (magenta) and TH (green) staining in the SN of mice. Note that the hyaluronan matrix is more profuse in the *pars compacta*, characterized by densely packed array of TH-positive neurons. **b.** Detail of HABP staining in the *pars compacta* (SNc) and *pars reticulata* (SNr). The hyaluronan matrix in the SNc resembles the interstitial matrix found in hippocampus and cortex, whereas in the SNr is present mostly in the perineuronal nets. **c.** Negative control experiment of Streptavidin-Atto647 labelling in the SN reveals effective blockage of endogenous biotins. Scale bars, 50 µm. Color bar represents fluorescence intensity.

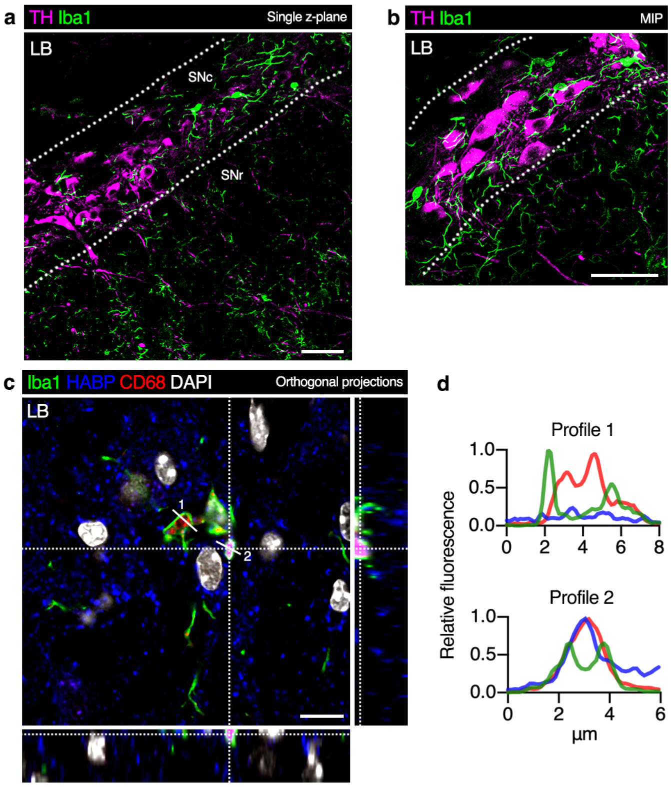

Supplementary Figure 6. Microglia in the SN after LB-induced neurodegeneration. *Related to Figure 4.* **a, b.** Confocal micrographs of the ventral midbrain showing the distribution of microglia in the SNc and SNr of LB-inoculated mice. Iba1-positive microglia occupy the void left by TH cells lost (**a**), but also surround surviving TH cells (**b**). In both cases, microglia invade the SNc, a region characterized by sparse Iba1 signal. Scale bar, 50 µm. **c.** Confocal images from the SN after LB-induced neurodegeneration, showing a microglial cell with HABP-negative (Profile 1) and HABP-positive (Profile 2) pockets. Both are positive for lysosomal marker CD68. Scale bar, 10 µm. **d.** Fluorescence intensity profiles from (**c**). Source data are provided as a Source Data file.

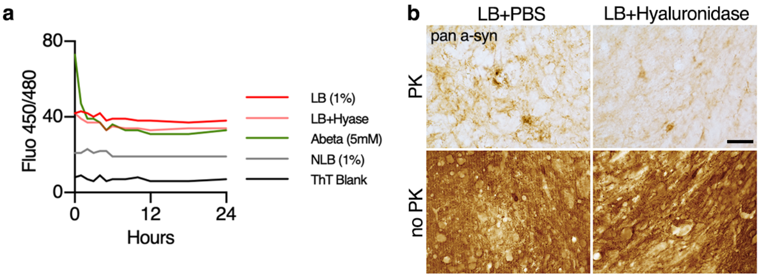

Supplementary Figure 7. Hyaluronidase does not alter LB *in vitro* but reduces alpha-synuclein pathology *in vivo*. *Related to Figure 5.* **a.** *In vitro* Thioflavin T assay shows absence of modification of the aggregation state of LB upon contact with Hyase. **b.** Proteinase-K assay on SN sections from LB-inoculated mice, 4 months after inoculation. Immunohistochemistry was performed with an alpha-synuclein antibody that recognizes both the rodent and human form. PK-resistant alpha-synuclein aggregates were observed mostly in the LB+PBS group, suggesting a modification of the pathology by Hyase at the time of inoculation. Scale bar, 50 µm. Source data are provided as a Source Data file.

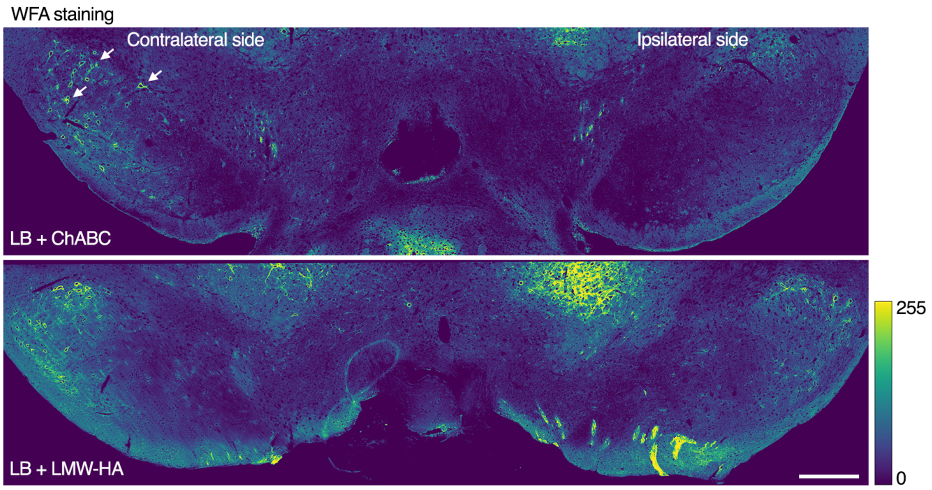

Supplementary Figure 8. Chondroitinase ABC removes CSPGs-based perineuronal nets (PNNs) *in vivo*. *Related to Figure 5.* Confocal tile scans showing Wisteria Floribunda Agglutinin (WFA)-staining in the SN 72hs after co-injection with LBs. Note the strong WFA signal in the PNNs on the contralateral side (white arrows), which is absent in the ChABC-injected side. Injection of LMW-HA is presented as control. Scale bar, 250 µm. Color bar represents fluorescence intensity.

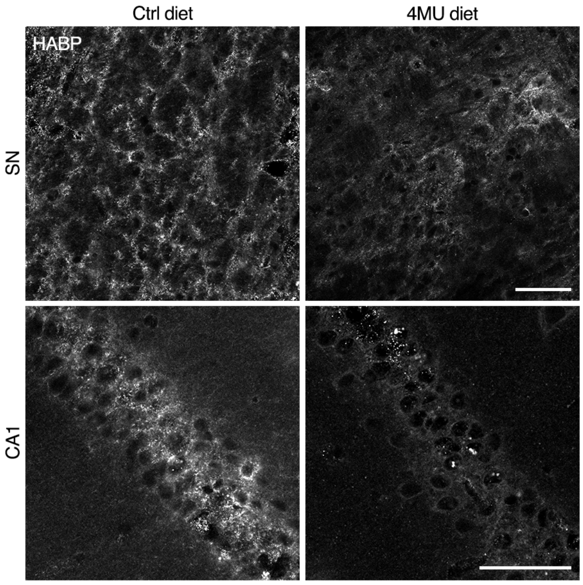

Supplementary Figure 9. Chronic 4MU treatment reduces hyaluronan content in mouse brain. *Related to Figure 6.* Confocal micrographs showing HABP labeling in SN (ventral midbrain) and CA1 (hippocampus) regions of mice after 1 month of hyaluronan-synthase inhibitor 4-methylumbelliferone (4MU) or normal diet. Note the patchy labelling in HA-depleted mice, compared to the profuse interstitial matrix observed in Ctrl. Scale bar, 50 µm.

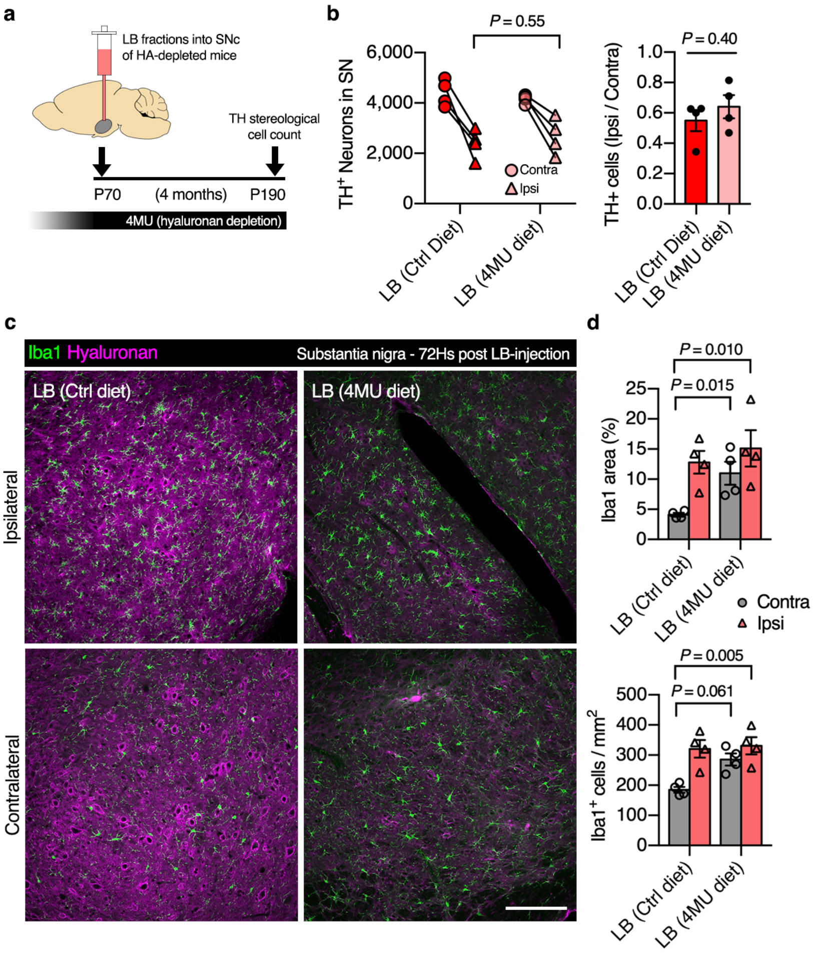

Supplementary Figure 10. Chronic hyaluronan depletion does not alter LB-induced neurodegeneration in mice but induces widespread microgliosis. *Related to Figure 6.* **a**. Experimental timeline of LB mouse model in a context of HA chronic depletion. **b.** TH stereology revealed a tendency to decrease LB-induced dopaminergic cell loss after 4 months, in mice administered with 4MU vs control, evidenced by TH-positive cell count and ipsilateral/contralateral TH-positive cell ratio (Two tailed Student's *t* test, *n* = 4 mice). **c.** Iba1 staining was unexpectedly increased in the contralateral side of 4MU+LB mice, indicating persistent microgliosis after 1 month of chronic HA depletion. Scale bar, 50 µm. **d.** Quantification of Iba1 levels and Iba1-positive cell somas in the SN of LB-inoculated mice fed with control and 4MU diet. (two-way ANOVA with Holm-Sidak's post-hoc test, *n* = 4 mice. Error bars represent mean ± SEM. Source data are provided as a Source Data file.

Supplementary Table 1. Primer sequences used for qPCR

Gene name	Genbank	FW	RV
cd3e	NM_007648	TGCCTCCTCTGCTAGTAGCCA	TGAGCAGCCTGATTCTTTCAAAG
cd44	NM_009851	AGGAGAAGCCAACAGAAATGAAA	TGTACATCACACCTCCTCCTCGT
cd68	BC021637.1	ACCCATCCCCACCTGTCTCT	TGATGTAGGTCCTGTTTGAATCCA
gapdh	NM_008084	TCAAGAAGGTGGTGAAGCAG	TGGGAGTTGCTGTTGAAGTC
gfap	NM_010277	AGGTCCGCTTCCTGGAACA	GGGCTCCTTGGCTCGAA
has1	NM_008215	AGGGCTCTTAAAGGAGGAGTCC	AGAAGGTAAACTGAGTCCCCAGAA
has2	NM_008216	CAAAGAGGTTCGTTCAAGTTCTGA	TGTGTTTGTTTCCCACTAGCTCTC
has3	NM_008217	CTGGTCTATCTCCTCCAACAGCTT	GCTGGGATAATGAAGAGCTACAGAA
hmmr	NM_013552	GCAGGCCTTTTACTCAGATCTCC	GCCATGTGATGCTTGCCTTAA
hyal1	NM_008317	GTGCCAAGCCCTATGCTAATAAG	GCATGTCCATTGCAAAGACTGA
hyal2	NM_010489	GTCCCACATACACCCGAGGA	GGCACTCTCACCGATGGTAGA
hyal3	NM_178020	GGACGACCTGATGCAGACTATTG	GGTCCCCCCAGAGTACCACT
icam1	NM_010493	TGCTCCTCCACATCCTGGA	GGCATGGTGGCTGACATTG
il1b	NM_008361	TCGCTCAGGGTCACAAGAAA	TCAGAGGCAAGGAGGAAAACAC
il6	NM_031168	TACTCGGCAAACCTAGTGCGT	ATTTTCTGACCACAGTGAGGAATG
itgam (CR3)	NM_001082960	CTCATCACTGCTGGCCTATACAA	GCAGCTTCATTCATCATGTCCTT
ptprc (CD45)	NM_011210	TGGGACAACGCAGACTCTCA	CTGCACAGCCATGTTCTTTCAT
tlr2	NM_011905	TGTCTCCACAAGCGGGACTT	CTCTTTTCGATGGAATCGATGAT
tlr4	NM_021297	GGACATGCCTTGTGAGATGGAT	CATGCTGACTGAAATAAGGTGAAAG
tnfa (TNF-α)	NM_013693	GCCTCTTCTCATTCCTGCTT	TGGGAACTTCTCATCCCTTT
trem2	NM_031254	GGCACCAACTTCAGATCCTCAC	GCTGTAGTTCTCCTCCCACTCAGA
ubc	NM_019639	AGACAGACGTACCTTCCTCACCA	CCCATCACACCCAAGAACAAG
