# Supplementary material for: Synucleinopathy alters nanoscale organization and diffusion in the brain extracellular space through hyaluronan remodeling
Source: Nat Commun. 2020 Jul 10;11:3440. doi: 10.1038/s41467-020-17328-9 (PMC7351768; doi:10.1038/s41467-020-17328-9)
Supplement: Supplementary file 3 — Description of Additional Supplementary Information [file 41467_2020_17328_MOESM3_ESM.pdf]

Descriptions of Additional Supplementary Files

Supplementary Movie 1

Description: SWCNT diffusion is effectively constrained in adult brain ECS. Near-infrared (NIR) videos of SWCNT at 37ºC, freely diffusing in artificial cerebro-spinal fluid (left) or constrained by the brain ECS after in vivo inoculation (right).

Supplementary Movie 2

Description: Long-term NIR acquisition of multiple SWCNTs in the substantia nigra of parkinsonian mice. SWCNTs have exceptional brightness and photostability, enabling high signal-to-noise ratio and extended tracking. Since SWCNTs diffusion occurs in a tridimensional space, they occasionally go out-of-focus along their trajectories. Only focused trajectory portions are analyzed, as independent tracks in different regions. Although SWCNTs appear to be confined to a region, they eventually move out of the field of view to a different area, which limits the length of acquisitions.

Supplementary Movie 3

Description: SWCNT diffusion is less constrained in the pathological brain. NIR movies of SWCNTs diffusing in the brain ECS in acute slices from control mice (left) or mice with LB-induced neurodegeneration (right).

Supplementary Movie 4

Description: SWCNT diffusion in the brain is less constrained after chronic hyaluronan depletion. NIR movies of SWCNTs diffusing in the brain ECS in acute slices from mice fed with control diet (left) or diet containing Has inhibitor 4MU (right).
