# Supplementary material for: Synucleinopathy alters nanoscale organization and diffusion in the brain extracellular space through hyaluronan remodeling
Source: Nat Commun. 2020 Jul 10;11:3440. doi: 10.1038/s41467-020-17328-9 (PMC7351768; doi:10.1038/s41467-020-17328-9)
Supplement: Supplementary file 8 — Reporting Summary [file 41467_2020_17328_MOESM8_ESM.pdf]

nature research

Corresponding author(s): Erwan Bezard, Laurent Cognet

Last updated by author(s): Jun 10, 2020

Reporting Summary

Nature Research wishes to improve the reproducibility of the work that we publish. This form provides structure for consistency and transparency in reporting. For further information on Nature Research policies, see Authors & Referees and the Editorial Policy Checklist .

Statistics

For all statistical analyses, confirm that the following items are present in the figure legend, table legend, main text, or Methods section.

n/a	Confirmed	
☐	☒	The exact sample size (*n*) for each experimental group/condition, given as a discrete number and unit of measurement
☐	☒	A statement on whether measurements were taken from distinct samples or whether the same sample was measured repeatedly
☐	☒	The statistical test(s) used AND whether they are one- or two-sided<br>*Only common tests should be described solely by name; describe more complex techniques in the Methods section.*
☐	☒	A description of all covariates tested
☐	☒	A description of any assumptions or corrections, such as tests of normality and adjustment for multiple comparisons
☐	☒	A full description of the statistical parameters including central tendency (e.g. means) or other basic estimates (e.g. regression coefficient) AND variation (e.g. standard deviation) or associated estimates of uncertainty (e.g. confidence intervals)
☐	☒	For null hypothesis testing, the test statistic (e.g. *F*, *t*, *r*) with confidence intervals, effect sizes, degrees of freedom and *P* value noted<br>*Give P values as exact values whenever suitable.*
☒	☐	For Bayesian analysis, information on the choice of priors and Markov chain Monte Carlo settings
☒	☐	For hierarchical and complex designs, identification of the appropriate level for tests and full reporting of outcomes
☒	☐	Estimates of effect sizes (e.g. Cohen's *d*, Pearson's *r*), indicating how they were calculated

Our web collection on statistics for biologists contains articles on many of the points above.

Software and code

Policy information about availability of computer code

Data collection	NIR images were acquired with LightField (Teledyne) Confocal images were acquired with LAS X (Leica). EM ultramicrographs were acquired with DigitalMicrograph (Gatan). Immunohistochemistry images were acquired with NIS Elements (Nikon). TH cell counts were done in Mercator (ExploraNova).
Data analysis	EM and immunohistochemical data was analyzed in Fiji/ImageJ (NIH). NIR images were analyzed with MetaMorph (Molecular Devices) for initial coordinates detection and MATLAB for all subsequent superresolution analysis. Excel 2013 (Microsoft) was used to sort and normalize fluorescence data. qPCR data was analyzed with Gene Expression Analysis Software Environment (INSERM). ThT and ELISA were analyzed with Mars v3.01 R2 (BMG Labtech). MATLAB was used for correlation analysis and KS test of local thickness data. GraphPad 7.0 (Prism) was used for all other statistical analysis.

For manuscripts utilizing custom algorithms or software that are central to the research but not yet described in published literature, software must be made available to editors/reviewers. We strongly encourage code deposition in a community repository (e.g. GitHub). See the Nature Research guidelines for submitting code & software for further information.

Data

Policy information about availability of data

All manuscripts must include a data availability statement. This statement should provide the following information, where applicable:

- Accession codes, unique identifiers, or web links for publicly available datasets
- A list of figures that have associated raw data
- A description of any restrictions on data availability

The data that support the findings of this study are available from the corresponding authors upon request.

Field-specific reporting

Please select the one below that is the best fit for your research. If you are not sure, read the appropriate sections before making your selection.

- [x] Life sciences
- [ ] Behavioural & social sciences
- [ ] Ecological, evolutionary & environmental sciences

For a reference copy of the document with all sections, see nature.com/documents/nr-reporting-summary-flat.pdf

Life sciences study design

All studies must disclose on these points even when the disclosure is negative.

Sample size	No statistical method was used to decide sample sizes. Since we use previously reported methodologies routinely used in our laboratories, we determined sample size from our own experience (refs 2, 18, 19) and other publications in the field. In the case of cryofixation-EM experiments, 4MU and Hyase experiments, we determined sample size on the base of pilot studies performed on naïve mice of similar age, gender and strain.
Data exclusions	We discarded SWCNT trajectories that rendered a plateau-shaped global MSD as we considered these nanotubes as immobile. We discarded EM images with clear freezing artifacts that rendered membranes (and therefore ECS) undiscernible. In qPCR analysis, we discarded genes with high CT (>35) and reported as "not detected". In IF analysis, we discarded sections that were less than 100 microns from the point of injection, to exclude inflammation due to mechanical damage. No other data points were excluded from the analysis.
Replication	Methodologies that are routinely used in our laboratories were successfully reproduced according to our previous publications (LB-induced neurodegeneration, local width and diffusivity values from single-nanotube tracking, IF stainings and quantifications). For newly acquired methodologies (cryofixation-EM, 4MU treatment, Hyase and ChABC injection), we conducted pilot experiments were we validated those methods, which were successfully replicated in the study. The key finding of increased ECS width and diffusion in LB-inoculated mice was replicated in two completely different experiments performed independently that achieved the same conclusion
Randomization	Mice were selected randomly for all experiments.
Blinding	In single-nanotube tracking, cryofixation-EM analysis and stereological cell counts, the experimenter was blind to the experimental group. For all other experiments, analysis was not blinded. Nevertheless, all image analysis protocols (except for phagocytosis quantification) were semi-automated routines developed prior to data acquisition.

Reporting for specific materials, systems and methods

We require information from authors about some types of materials, experimental systems and methods used in many studies. Here, indicate whether each material, system or method listed is relevant to your study. If you are not sure if a list item applies to your research, read the appropriate section before selecting a response.

Materials & experimental systems

n/a	Involved in the study	
[ ]	[x]	Antibodies
[x]	[ ]	Eukaryotic cell lines
[x]	[ ]	Palaeontology
[ ]	[x]	Animals and other organisms
[x]	[ ]	Human research participants
[x]	[ ]	Clinical data

Methods

n/a	Involved in the study	
[x]	[ ]	ChIP-seq
[x]	[ ]	Flow cytometry
[x]	[ ]	MRI-based neuroimaging

Antibodies

Antibodies used	Hyaluronic Acid Binding Protein biotinylated (5 µg/ml, Merck Millipore, catalog number 385911); Wisteria Floribunda Agglutinin biotynilated (1:200, Vector Labs, catalog number B-1355), Iba1 (1:500, Wako, catalog number 019-19741); CD68 clone FA11 (1:100, Bio-Rad, catalog number MCA1957); TH clone LCN1 (1:2000 IF, 1:5000 IHC, Merck-Millipore, catalog number MAB318); human alpha-synuclein clone Syn211 (1:1000, ThermoFisher, catalog number MA5-12272); alpha-synuclein clone 42 (1:5000, BD Biosciences, catalog number 610787); Streptavidin-Atto647N (1:500, Sigma-Aldrich, catalog number 94149); goat anti-mouse IgG Alexa488 (1:500, ThermoFisher, catalog number A-11001); goat anti-mouse IgG Alexa594 (1:500, ThermoFisher, catalog number A-11032); goat anti-rabbit IgG Alexa488 (1:500, ThermoFisher, catalog number A-11008); goat anti-rat IgG Alexa594 (1:500, ThermoFisher, catalog number A-11007).
Validation	All antibodies are commercially available, and validated for the applications used in this study. Validation information is available on the manufacturer's publicly accessible datasheets.

Animals and other organisms

Policy information about studies involving animals; ARRIVE guidelines recommended for reporting animal research

Laboratory animals	Male C57BL6/J adult (+P70) mice purchased from Charles River (France) were used for all experiments. For 4MU treatments, we started diet in P50 animals, in order to achieve effective hyaluronan depletion by P70, when LB were inoculated. No transgenic mice were used in this study.
Wild animals	No wild animals were used in this study.
Field-collected samples	No field-collected samples were used in this study.
Ethics oversight	The Institutional Animal Care and Use Committee of Bordeaux (CE50) and the Ministère de l'Enseignement supérieur, de la Recherche et de l'Innovation (France) approved experiments under licenses #5520-2016052514328805 (LB and Hyase experiments) and #10721-2017071213284522 (4MU experiments).

Note that full information on the approval of the study protocol must also be provided in the manuscript.
